# Wavelet Element Modelling for Inviscid Fluid–Solid Coupling Problem based on Partitioned Approach

**DOI:** 10.3390/ma13173699

**Published:** 2020-08-21

**Authors:** Zhi-Bo Yang, Hao-Qi Li, Bai-Jie Qiao, Xue-Feng Chen

**Affiliations:** 1 School of Mechanical Engineering, Xi’an Jiaotong University, Xi’an 710049, China; lihaoqi2014@stu.xjtu.edu.cn (H.-Q.L.); qiao1224@xjtu.edu.cn (B.-J.Q.); chenxf@mail.xjtu.edu.cn (X.-F.C.); 2 The State Key Laboratory for Manufacturing Systems Engineering, Xi’an 710049, China

**Keywords:** wavelet element, fluid–solid coupling, partitioned approach, wave propagation

## Abstract

To provide a simple numerical formulation based on fixed grids, a wavelet element method for fluid–solid modelling is introduced in this work. Compared with the classical wavelet finite element method, the presented method can potentially handle more complex shapes. Considering the differences between the solid and fluid regions, a damping-like interface based on wavelet elements is designed, in order to ensure consistency between the two parts. The inner regions are constructed with the same wavelet function in space. In the time and spatial domains, a partitioned approach based on Jacobi iteration is combined with the pseudo-parallel calculation method. Numerical convergence analyses show that the method can serve as an alternative choice for fluid–solid coupling modelling.

## 1. Introduction

In the context of structural health monitoring, as well as seismic and acoustic exploration, the numerical modelling and analyses of fluid–solid interfaces and coupling have been considered crucial issues, due to the complexity of the related physical phenomena. For waves propagating in a single medium (i.e., fluid or solid), nearly all types of numerical methods can be used, such as the finite difference method [1,2] and its derivative formulations [3], boundary element methods [4], spectral element methods [5,6], pseudo-spectral element methods (i.e., time–domain spectral element methods) [7], wavelet spectral methods [8,9,10], and mixed formulations based on spectral methods [11]. Although the performances of these numerical methods typically differ, due to the corresponding superiorities or shortcomings, reasonable results can be usually obtained for the self-contained establishment of modelling and solving methodologies. Fluid–solid interfaces lead to significant numerical difficulties, as the correct implementation of physical matching conditions plays an important role in the convergence of numerical algorithms. Modelling such an interface involves many issues concerning the selection of grid techniques, simulation techniques, and time discretization.

In fluid–solid interaction problems, both the fluid domain and the solid domain are deformed. For elastic and incompressible problems, the solid region is usually modelled by a fixed grid technique, due to its small deformation, while the grid of the fluid part can be altered between moving and fixed grids [12]. The arbitrary Lagrangian–Eulerian formulation [13] is a typical moving grid formulation, in which the grid can move with arbitrary velocity; but not necessarily at the velocity of the fluid. The movement of the grid involves interpolation from the old to the new grid, inevitably causing errors. Moreover, the interaction between the fixed solid grid and the moving fluid grid is also complicated, although the arbitrary Lagrangian–Eulerian formulation has the advantage that the wall shear stress at the fluid–structure interface can be calculated accurately. To obtain a simpler formulation for the fluid–solid coupling problem, a fixed grid is used for the fluid. The immersed boundary method developed by Peskin [14] limits the solid by acting like fibers containing a chain of solid nodes. An immersed fiber can occupy no volume in the fluid domain in the original version. The numerical properties of the immersed boundary method mean that it is straightforward to determine the type of the boundary conditions for the fluid grid, if the fluid surrounds the structure, while some non-physical boundary conditions are required for the interface. Another fixed grid technique is the fictitious domain method. The distributed Lagrange multiplier fictitious domain method was proposed by Glowinski et al. [15] for the interaction between solids and fluids. In the formulation of this method, the solid region immersed in the fluid part is filled with the same fluid as the remainder of the fluid domain. Additional Lagrange multipliers are used to impose the equality of speed for the fictitious fluid and the solid part. To further simplify the formulation, the presented work used wavelet element modelling for both solid and fluid parts, with a fixed boundary. With the same numerical formulation, the interface can be designed and assembled.

Besides grid techniques (i.e., spatial discretization), simulation techniques and solving techniques are other aspects to be considered. The fluid–solid interaction problem can be implemented in a monolithic or partitioned formulation [16]. The monolithic approach solves the flow equations and the structural equations simultaneously and, thus, the interaction can be considered during the solution process. For a fixed grid, following the monolithic approach means using a set of larger matrices, which contain the solid, fluid, and the coupling, which are constructed and involved in every time step, while the stability of the calculation can be ensured in this process [17]. In contrast, partitioned techniques allow for solving the flow equations separately from the structural equations, and vice versa [18]. To transfer and update data during computation, the interface is important. The main advantage of the partitioned approach is the reuse of reliable and optimized codes for flow equations and structural equations. This property allows for the flexible assembly of algorithms. The large family of finite element methods provides the possibility of addressing this issue. The wavelet element method has been shown to be an efficient alternative tool for modelling wave motion [19], but the absence of a Jacobian matrix, interface modelling, and the solving methodology for decoupling limits its application for the considered issue. In addition, a large number of iterations in the time and space domains requires efficient solving in every step. Thus, the inverse of the total stiffness should be avoided, and an indirect path to solve the variable in both fluid and solid should be implemented.

In this work, we developed an isoparametric wavelet element formulation with Jacobian matrix, which allows the method to potentially handle more complex shapes. With the consideration of the balance between physical and geometric interpolation, a B-spline-based wavelet is used as the interpolating/shape function. Different with the classical interpolating/shape function, the transform matrix is introduced to avoid the use of wavelet coefficient and controlling point for B-spline-based wavelets. The superiorities of B-spline in structural analysis and geometric approximation make the corresponding wavelet element a promising selection for the inviscid fluid–solid coupling problem. Considering the difference between solid and fluid regions, in terms of their degrees of freedom, a damping-like interface based on wavelet elements is designed. The inner regions, both for the fluid and the solid, are constructed using the same wavelet function in the spatial domain. A partitioned approach based on Jacobi iteration [12] is used, combined with the pseudo-parallel calculation method to address the “out of memory” problem.

## 2. Formulation of the Inspected Problem

### 2.1. The Strong Form (Physical Models)

In order to simplify the analysis and to highlight the performance of the presented numerical process, in this paper, the fluid part is considered inviscid and incompressible, while the solid part is considered elastic and isotropic. The strong form (the partial differential equation) of the wave motion equation in the solid region can be written, in vector form, as Equations (1)–(3) based on Lame parameters:

(1) $$\rho_{s}\ddot{u}=\nabla \cdot \sigma +f_{s},$$

(2) σ=Dε=λtr(ε)I+2με,

(3) $$\varepsilon =\frac{1}{2}\left[\nabla u+{\left(\nabla u\right)}^{T}\right],$$

where vector ***u*** denotes the displacement of the solid. For the two-dimensional case, ***u*** is expressed by [*u_x_*, *u_y_*]^T^. The symbols ***σ*** and ***ε*** denote the stress and strain tensors, respectively. The symbols *λ* and *μ* are the Lame parameters, and *ρ_s_* is the mass density of the solid waveguide, referring to the inertial force of the inspected region. The matrix **I** is the identity tensor. The vector ***f_s_*** defines the general external force in the solid region. The function tr(·) is employed to obtain the trace of the bracketed term. The fluid region, where only the acoustic wave propagates, is governed by the conservation and dynamics equations:

(4) $$\rho_{f}\dot{v}+\nabla p=0,$$

(5) $$\dot{p}+\rho c^{2}\nabla \cdot v=0,$$

where the vector ***v*** denotes the velocity of the fluid, the constant $c=\sqrt{k/\rho_{f}}$ is the velocity of the acoustic wave, and *k* is the bulk modulus of fluid. The mass density of fluid is defined by *ρ_f_*.

As the fluid is irrotational, the variable ***v*** is replaced by the gradient of the potential function φ. Substituting the relationship v=∇φ into Equations (4) and (5), a second order system which only involves the potential function variable can be obtained:

(6) $$\frac{1}{c^{2}}\ddot{\varphi }=\nabla^{2}\varphi .$$

The above equations independently define the dynamic properties in the solid and fluid. To couple the two waveguides together, we need to find the connection between ***u*** and ***v***. To address this problem, Komatitsch et al. [18] proposed a fluid–solid interface method. From Equation (4), it can be obtained as follows:

(7) $$-\rho_{f}\nabla \dot{\varphi }=\nabla p\Rightarrow -\rho_{f}\dot{\varphi }=p.$$

Considering Equation (7) and the continuity of traction on the interface (the boundary between solid and fluid), τ=σ⋅n (where ***n*** denotes the unit normal to the interface), we obtain the following:

(8) $$\tau =\rho_{f}\dot{\varphi }\cdot n.$$

The continuity of the normal component of speed is expressed as:

(9) $$n\cdot \nabla \varphi =n\cdot \dot{u}.$$

Note that v=∇φ. Equation (9) constructs the energy transport path between solid and fluid.

In the present model, it should be noticed that the shear force is considered in the solid region but absent in the fluid region. The present interface fully ignores the viscosity on the acoustic wave propagation, which is not an accurate model as the equilibrium condition on the interface is not satisfied due to neglection of shear stress. The balance of tangential element of the stress on the interface is ignored. A more precise interface containing the shear effect should be considered to achieve more accurate results in practice.

### 2.2. The Weak Form (Numerical Models)

In finite element methods, problems are solved using their corresponding weak forms. By dotting the partial differential equation with the trial function (or interpolating function) ψ = [ψ*_x_*(*x*, *y*), ψ*_y_*(*x*, *y*)]^T^ for the solid region, and ψ(*x*, *y*) for the fluid region, and integrating by parts over the field of interest, we obtain Equation (10) for the solid region:

(10) $$\int_{\Omega_{s}}\psi \rho_{s}\ddot{u}d\Omega +\int_{\Omega_{s}}\nabla \psi D\nabla ud\Omega -\int_{\Gamma }\psi \rho_{f}n\dot{\varphi }d\Gamma =\int_{\Omega_{s}}\psi f_{s}d\Omega ,$$

and Equation (11) for the fluid region:

(11) $$\int_{\Omega_{f}}\frac{1}{c^{2}}\psi \ddot{\varphi }d\Omega +\int_{\Omega_{f}}\nabla \psi \nabla \varphi d\Omega +\int_{\Gamma }\psi n\dot{u}d\Gamma =0,$$

where the inspected solid and fluid regions are denoted by Ω*_s_* and Ω*_f_*, respectively, and the interface between them is defined by the symbol Γ. In the above equations, we consider a wave source, ***f****_s_*, in the solid region, which can be implemented into the fluid region. Based on the interface components in Equations (10) and (11), the solid and the fluid system are coupled. In numerical formulation, the Dirichlet–Neumann decomposition method is used to connect the coupled regions:

(12) $$\varphi =\sqrt{{\dot{u}}_{x}^{2}+{\dot{u}}_{y}^{2}}\;(\mathrm{from}\;\mathrm{solid}\;\mathrm{to}\;\mathrm{fluid}),$$

by which the velocities in the solid part can be converted to the velocity of the fluid. By

(13) $$\dot{u}={\left\{n_{x}\nabla \varphi ,n_{y}\nabla \varphi \right\}}^{T}\;(\mathrm{from}\;\mathrm{fluid}\;\mathrm{to}\;\mathrm{solid}),$$

the velocity vector in the solid can be calculated from the variables in the fluid part.

### 2.3. Wavelet Element Spatial Discretization

Numerical solution of the wave propagation in solid–fluid coupling problems involves the discretization of spatial and temporal domains. We chose the *m*th-order *j* scale B-spline wavelets on the interval (BSWI) interpolating function $\psi_{m,k}^{j}(\xi )$ for spatial discretization. The region Ω, either for the solid or fluid, is divided into a set of non-overlapping sub-domains, $\Omega_{e}$, following which each sub-domain is mapped onto a unit interval, considering the dimension of the problem being analyzed. According to the *m*th-order 0 scale B-spline functions and the corresponding wavelets given by Goswami [20], the *j* scale *m*th-order BSWI scaling functions $\psi_{m,k}^{j}(\xi )$, denoted by BSWI*m_j_*, can be derived as:

(14) $$\begin{array}{cc} \psi_{m,k}^{j}(\xi )= & \left\{ \begin{array}{l} \psi_{m,k}^{\;l}(2^{j-l}\xi ),\;k=-m+1,\ldots ,-1\\ \psi_{m,2^{j}-m-k}^{\;l}(1-2^{j-l}\xi ),\;k=2^{j}-m+1,\ldots ,2^{j}-1\;\\ \psi_{m,0}^{\;l}(2^{j-l}\xi -2^{-l}k),\;k=0,\ldots ,2^{j}-m \end{array}\right.\begin{array}{l} \left(0\;\mathrm{boundary}\;\mathrm{scaling}\;\mathrm{functions}\right)\\ \left(1\;\mathrm{boundary}\;\mathrm{scaling}\;\mathrm{functions}\right)\\ \left(\mathrm{inner}\;\mathrm{scaling}\;\mathrm{functions}\right) \end{array} \end{array}.$$

Based on Equation (14), the interpolating functions, in horizontal and vertical directions, are defined as:

(15) $$\begin{array}{c} \psi_{\xi }=\left\{\;\psi_{m,-m+1}^{j}(\xi )\;\psi_{m,-m+2}^{j}(\xi )\ldots \psi_{m,2^{j}-1}^{j}(\xi )\right\},\\ \psi_{\eta }=\left\{\;\psi_{m,-m+1}^{j}(\eta )\;\psi_{m,-m+2}^{j}(\eta )\ldots \psi_{m,2^{j}-1}^{j}(\eta )\right\}, \end{array}$$

where *ξ* and *η* are restricted to the interval [0, 1], respectively, depicting the normalized *x* and *y* co-ordinates. The two-dimensional interpolating function is formulated based on the Kronecker product between the two vectors in Equation (15); namely, $\Psi =\psi_{\xi }⊗\psi_{\eta }$. In the classical finite element method, the unknown field function *u* is approximated by the interpolating function **N** and node vectors ***u****^e^* as:

(16) $$u^{e}(\xi ,\eta ,t)=\sum_{i}^{n+1}\sum_{j}^{n+1}N_{i}(\xi )N_{j}(\eta )u^{e}(\xi_{i},\eta_{j},t)=Nu^{e}.$$

Note that the wavelet coefficients can be obtained when the interpolating function **N** is replaced by Ψ. The physical meaning of the wavelet coefficients is not evident, due to the overlap in support, and the boundary condition is not easy to implement. Therefore, an additional matrix **T** is required to transform the wavelet coefficients into the physical domain, in which the interpolating function **N** yields:

(17) ΨT=N,

where the transform matrix is $\begin{array}{c} T={\left\{\psi_{\xi }^{T}(\xi_{1}),\;\psi_{\xi }^{T}(\xi_{2})\;\ldots \;\psi_{\xi }^{T}(\xi_{n+1})\right\}}^{-T}⊗{\left\{\psi_{\eta }^{T}(\eta_{1}),\;\psi_{\eta }^{T}(\eta_{2})\;\ldots \;\psi_{\eta }^{T}(\eta_{n+1})\right\}}^{-T} \end{array}$. Equations (10) and (11) can be rewritten as:

(18) $$\{\begin{array}{ll} \sum_{e}\int_{\Omega_{s}^{e}}{\left(\Psi Tu^{e}\right)}^{T}\rho_{s}\Psi T{\ddot{u}}^{e}d\Omega +\sum_{e}\int_{\Omega_{s}^{e}}\nabla {\left(\Psi Tu^{e}\right)}^{T}D\nabla \Psi Tu^{e}d\Omega & -\sum_{e}\int_{\Gamma_{}^{e}}{\left(\Psi Tu^{e}\right)}^{T}\rho_{f}n\Psi T{\dot{\varphi }}^{e}d\Gamma =\sum_{e}\int_{\Omega_{s}^{e}}{\left(\Psi Tu^{e}\right)}^{T}f_{s}d\Omega \\ \sum_{e}\int_{\Omega_{f}^{e}}\frac{1}{c^{2}}{\left(\Psi T\varphi^{e}\right)}^{T}\varphi^{e}\Psi T{\ddot{\varphi }}^{e}d\Omega +\sum_{e}\int_{\Omega_{f}^{e}}\nabla {\left(\Psi T\varphi^{e}\right)}^{T}\nabla \Psi T\varphi^{e}d\Omega & +\sum_{e}\int_{\Gamma^{e}}{\left(\Psi T\varphi^{e}\right)}^{T}n{\dot{u}}^{e}d\Gamma =0 \end{array},$$

where the superscript *e* depicts “elemental”. Equilibrium is also satisfied in each element. Based on the Hamilton principle, we further obtain the wavelet element method in matrix form:

(19) $$\{\begin{array}{l} \sum_{e}M_{s}^{e}{\ddot{u}}^{e}+\sum_{e}K_{s}^{e}u^{e}-\sum_{e}C_{s}^{e}{\dot{\varphi }}^{e}=\sum_{e}f_{s}{}^{e}\\ \sum_{e}M_{f}^{e}{\ddot{\varphi }}^{e}+\sum_{e}K_{f}^{e}\varphi^{e}+\sum_{e}C_{f}^{e}{\dot{u}}^{e}=0 \end{array}.$$

Assembling the elementary matrices together, we further obtain:

(20) $$\{\begin{array}{l} M_{s}^{}\ddot{u}+K_{s}^{}u-C_{s}^{}\dot{\varphi }=f_{s}{}^{}\\ M_{f}^{}\ddot{\varphi }+K_{f}^{}\varphi +C_{f}^{}\dot{u}=0 \end{array},$$

where **M** is the mass matrix and **K** is the stiffness matrix. The pseudo-damping **C** is the coupling matrix of the interface, which acts on the first order temporal derivatives of ***u*** and φ. It differs from the real damping, in that the energy is not dissipated in the layer but transported to the coupled region instead. The interface is integrated into the finite element formulation as a pseudo-damping layer, which couples the solid and fluid parts. The explicit forms of the corresponding global matrices for the two-dimensional case are:

(21) $$M_{s}^{}=\sum_{e}^{}\sum_{i}^{n+1}\sum_{j}^{n+1}w_{i}w_{j}{\left(\Psi T\right)}^{T}\rho_{s}\Psi T\det(J),$$

(22) $$M_{f}^{}=\sum_{e}^{}\sum_{i}^{n+1}\sum_{j}^{n+1}w_{i}w_{j}{\left(\Psi T\right)}^{T}\frac{1}{c^{2}}\Psi T\det(J),$$

(23) $$K_{s}=\sum_{e}^{}\sum_{i}^{n+1}\sum_{j}^{n+1}w_{i}w_{j}\nabla {\left(\Psi T\right)}^{T}D\nabla \Psi T\det(J),$$

(24) $$K_{f}=\sum_{e}^{}\sum_{i}^{n+1}\sum_{j}^{n+1}w_{i}w_{j}\nabla {\left(\Psi T\right)}^{T}\nabla \Psi T\det(J),$$

(25) $$C_{s}=\sum_{e}^{}\sum_{i}^{n+1}\sum_{j}^{n+1}w_{i}w_{j}{\left(\Psi T\right)}^{T}\rho_{f}n\Psi T\det(J),$$

(26) $$C_{f}=\sum_{e}^{}\sum_{i}^{n+1}\sum_{j}^{n+1}w_{i}w_{j}{\left(\Psi T\right)}^{T}n\Psi T\det(J),$$

where **J** is the Jacobian matrix which transforms the normal region to a curved region, and the constants *w_i_* and *w_j_* are the weights referring to the Gaussian integral. As an isoparametric method, the shape function related to **J** is also defined by the same BSWI used for interpolation. Here, we give the explicit expression of the Jacobian matrix based on BSWI4_3_ (the scale *j =* 3*,* and fourth order) element.

The even order spline is usually used in structural analysis; thus, the fourth-order spline is selected. In order to ensure the existence of inner wavelet, scale *j* and order *m* should yield:

(27) $$2^{j}>2m-1.$$

Then scale *j* is fixed at 3.

The BSWI4_0_ is given by [20], then the BSWI4_3_ function $\psi_{\xi }=\left\{\;\psi_{4,-3}^{3}(\xi )\;\psi_{4,-2}^{3}(\xi )\ldots \psi_{4,7}^{3}(\xi )\right\}$ can be derived from Equation (14) as:

(28) $$\psi_{4,-3}^{3}(\xi )=\{\begin{array}{ll} 1-3\times (2^{3}\xi )+3\times {\left(2^{3}\xi \right)}^{2}-{\left(2^{3}\xi \right)}^{3} & \xi \subset [0,1/8]\\ 0, & \xi ⊄[0,1/8] \end{array},$$

(29) $$\psi_{4,-2}^{3}(\xi )=\{\begin{array}{ll} 3\times (2^{3}\xi )-\frac{9}{2}\times {\left(2^{3}\xi \right)}^{2}+\frac{7}{6}\times {\left(2^{3}\xi \right)}^{3} & \xi \subset [0,1/8]\\ 2-3\times (2^{3}\xi )+\frac{3}{2}\times {\left(2^{3}\xi \right)}^{2}-\frac{1}{4}\times {\left(2^{3}\xi \right)}^{3} & \xi \subset [1/8,1/4]\\ 0 & \xi ⊄[0,1/4] \end{array},$$

(30) $$\psi_{4,-1}^{3}(\xi )=\{\begin{array}{ll} \frac{3}{2}{\left(2^{3}\xi \right)}^{2}-\frac{11}{12}{\left(2^{3}\xi \right)}^{3} & \xi \subset [0,1/8]\\ -\frac{3}{2}+\frac{9}{2}(2^{3}\xi )-3\times {\left(2^{3}\xi \right)}^{2}+\frac{7}{12}{\left(2^{3}\xi \right)}^{3} & \xi \subset [1/8,1/4]\\ \frac{9}{2}-\frac{9}{2}(2^{3}\xi )+\frac{3}{2}{\left(2^{3}\xi \right)}^{2}-\frac{1}{6}{\left(2^{3}\xi \right)}^{3} & \xi \subset [1/4,3/8]\\ 0 & \xi ⊄[0,3/8] \end{array},$$

(31) $$\psi_{4,0}^{3}(\xi )=\{\begin{array}{ll} \frac{1}{6}{\left(2^{3}\xi \right)}^{3} & \xi \subset [0,1/8]\\ \frac{2}{3}-2\times (2^{3}\xi )+2\times {\left(2^{3}\xi \right)}^{2}-\frac{1}{2}\times {\left(2^{3}\xi \right)}^{3} & \xi \subset [1/8,1/4]\\ -\frac{22}{3}+10\times (2^{3}\xi )-4\times {\left(2^{3}\xi \right)}^{2}+\frac{1}{2}\times {\left(2^{3}\xi \right)}^{3} & \xi \subset [1/4,3/8]\\ \frac{32}{3}-8\times (2^{3}\xi )+2\times {\left(2^{3}\xi \right)}^{2}-\frac{1}{3}\times {\left(2^{3}\xi \right)}^{3} & \xi \subset [3/8,1/2]\\ 0 & \xi ⊄[0,1/2] \end{array},$$

(32) $$\begin{array}{c} \psi_{4,1}^{3}(\xi )=\psi_{4,0}^{3}(\xi -\frac{1}{8}),\;\psi_{4,2}^{3}(\xi )=\psi_{4,0}^{3}(\xi -\frac{1}{4}),\;\psi_{4,3}^{3}(\xi )=\psi_{4,0}^{3}(\xi -\frac{3}{8}),\\ \psi_{4,4}^{3}(\xi )=\psi_{4,0}^{3}(\xi -\frac{1}{2}), \end{array}$$

and

(33) $$\psi_{4,5}^{3}(\xi )=\psi_{4,-1}^{3}(1-\xi ),\;\psi_{4,6}^{3}(\xi )=\psi_{4,-2}^{3}(1-\xi ),\;\psi_{4,7}^{3}(\xi )=\psi_{4,-3}^{3}(1-\xi ).$$

Similarly, we can obtain $\psi_{\eta }=\left\{\;\psi_{4,-3}^{3}(\eta )\;\psi_{4,-2}^{3}(\eta )\ldots \psi_{4,7}^{3}(\eta )\right\}$ and $\Psi =\psi_{\xi }⊗\psi_{\eta }$. The present element is constructed as the isoparametric element, thus Ψ is also used as the shape function. The direct interpolating by wavelet function induces the solution in the forms of wavelet coefficient **a** like:

(34) Ψa=F(x,y),

where *F*(*x*, *y*) depicts the unknow function to be interpolated. Thus, **T** matrix is employed to connect wavelet coefficient **a** with physical region **u** as:

(35) Tu=a,

where $T={\left\{\psi_{\xi }^{T}(\xi_{1}),\;\psi_{\xi }^{T}(\xi_{2})\;\ldots \;\psi_{\xi }^{T}(\xi_{n+1})\right\}}^{-T}⊗{\left\{\psi_{\eta }^{T}(\eta_{1}),\;\psi_{\eta }^{T}(\eta_{2})\;\ldots \;\psi_{\eta }^{T}(\eta_{n+1})\right\}}^{-T}$, and the $\xi_{i}$, $\eta_{i}$ can be flexibly selected in the interval [0, 1]. As we mentioned, the interpolating function/shape function can be replaced by ΨT=N by aid of the **T** matrix. The Jacobian matrix:

(36) $$J=\left[\begin{array}{cc} \frac{\partial x}{\partial \xi } & \frac{\partial y}{\partial \xi }\\ \frac{\partial x}{\partial \eta } & \frac{\partial y}{\partial \eta } \end{array}\right]=\left[\begin{array}{cc} \frac{\partial \Psi }{\partial \xi } & \frac{\partial \Psi }{\partial \xi }\\ \frac{\partial \Psi }{\partial \eta } & \frac{\partial \Psi }{\partial \eta } \end{array}\right]Td.$$

Here the location is denoted by **d** = (**x**, **y**)^T^ to distinguish with deflection **u**. The main entries of **J** matrix are calculated by:

(37) $$\frac{\partial \Psi }{\partial \xi }=\frac{\partial \psi_{\xi }}{\partial \xi }⊗\psi_{\eta },\;\frac{\partial \Psi }{\partial \eta }=\psi_{\xi }⊗\frac{\partial \psi_{\eta }}{\partial \eta }.$$

The main components of $\frac{\partial \psi_{\xi }}{\partial \xi }$ are:

(38) $$\frac{\partial \psi_{4,-3}^{3}(\xi )}{\partial \xi }=\{\begin{array}{ll} -24+48\times (2^{3}\xi )-24\times {\left(2^{3}\xi \right)}^{2} & \xi \subset [0,1/8]\\ 0, & \xi ⊄[0,1/8] \end{array},$$

(39) $$\frac{\partial \psi_{4,-2}^{3}(\xi )}{\partial \xi }=\{\begin{array}{ll} 24-72\times (2^{3}\xi )+28\times {\left(2^{3}\xi \right)}^{2} & \xi \subset [0,1/8]\\ -24+24\times (2^{3}\xi )-6\times {\left(2^{3}\xi \right)}^{2} & \xi \subset [1/8,1/4]\\ 0 & \xi ⊄[0,1/4] \end{array},$$

(40) $$\frac{\partial \psi_{4,-1}^{3}(\xi )}{\partial \xi }=\{\begin{array}{ll} 24\times (2^{3}\xi )-22\times {\left(2^{3}\xi \right)}^{2} & \xi \subset [0,1/8]\\ 36-48\times (2^{3}\xi )+14\times {\left(2^{3}\xi \right)}^{2} & \xi \subset [1/8,1/4]\\ -36+24\times (2^{3}\xi )-4\times {\left(2^{3}\xi \right)}^{2} & \xi \subset [1/4,3/8]\\ 0 & \xi ⊄[0,3/8] \end{array},$$

(41) $$\frac{\partial \psi_{4,0}^{3}(\xi )}{\partial \xi }=\{\begin{array}{ll} 4\times {\left(2^{3}\xi \right)}^{2} & \xi \subset [0,1/8]\\ -16+32\times (2^{3}\xi )-12\times {\left(2^{3}\xi \right)}^{2} & \xi \subset [1/8,1/4]\\ 80-64\times (2^{3}\xi )+12\times {\left(2^{3}\xi \right)}^{2} & \xi \subset [1/4,3/8]\\ -64+32\times (2^{3}\xi )-8\times {\left(2^{3}\xi \right)}^{2} & \xi \subset [3/8,1/2]\\ 0 & \xi ⊄[0,1/2] \end{array},$$

(42) $$\frac{\partial }{\partial \xi }\psi_{4,1}^{3}(\xi )=\frac{\partial }{\partial \xi }\psi_{4,0}^{3}(\xi -\frac{1}{8}),\;\frac{\partial }{\partial \xi }\psi_{4,2}^{3}(\xi )=\frac{\partial }{\partial \xi }\psi_{4,0}^{3}(\xi -\frac{1}{4}),$$

(43) $$\frac{\partial }{\partial \xi }\psi_{4,3}^{3}(\xi )=\frac{\partial }{\partial \xi }\psi_{4,0}^{3}(\xi -\frac{3}{8}),\;\frac{\partial }{\partial \xi }\psi_{4,4}^{3}(\xi )=\frac{\partial }{\partial \xi }\psi_{4,0}^{3}(\xi -\frac{1}{2}),$$

and

(44) $$\begin{array}{c} \frac{\partial }{\partial \xi }\psi_{4,5}^{3}(\xi )=\frac{\partial }{\partial \xi }\psi_{4,-1}^{3}(1-\xi ),\;\frac{\partial }{\partial \xi }\psi_{4,6}^{3}(\xi )=\frac{\partial }{\partial \xi }\psi_{4,-2}^{3}(1-\xi ),\\ \;\frac{\partial }{\partial \xi }\psi_{4,7}^{3}(\xi )=\frac{\partial }{\partial \xi }\psi_{4,-3}^{3}(1-\xi ). \end{array}$$

$\frac{\partial \psi_{\eta }}{\partial \eta }$ is obtained in the same manner, and the **J** matrix can be calculated according to Equation (37).

**Figure 1:**
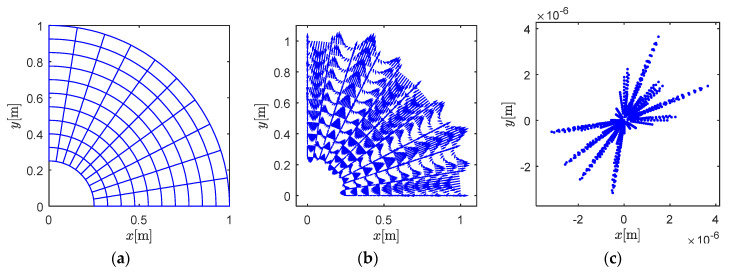
B-spline wavelets on the interval (BSWI)4_3_ interpolation for a quarter circle: (**a**) BSWI interpolation, (**b**) the quiver figure for error, (**c**) the value of error.

The interpolating results of a quarter circle idealized by one BSWI4_3_ element is presented in Figure 1. The element contains 11 × 11 inner nodes as shown in Figure 1a. Figure 1b,c present the distribution and values of error independently. The scattered points in Figure 1c illustrate the value of every arrow in Figure 1b. It is seen the interpolating error is limited to an acceptable level; with the use of more elements in modelling, the error can be further restrained.

### 2.4. Temporal Discretization and Partitioned Approach

The central difference time integration scheme for temporal discretization is Equation (45):

(45) $$\{\begin{array}{c} \underset{M_{s}^{0}}{\underset{︸}{\left(\frac{1}{\Delta t^{2}}M_{s}\right)}}u_{{}^{{}_{t+\Delta t}}}=f_{s}^{t}-\underset{{\hat{F}}_{s}}{\underset{︸}{K_{s}u_{t}}}+\underset{M_{s}^{1}}{\underset{︸}{\left(\frac{2}{\Delta t^{2}}M_{s}\right)}}u_{t}-\underset{M_{s}^{2}}{\underset{︸}{\left(\frac{1}{\Delta t^{2}}M_{s}\right)}}u_{{}^{{}_{t-\Delta t}}}+\underset{C_{s}^{0}}{\underset{︸}{\left(\frac{1}{2\Delta t}C_{s}\right)}}(\varphi_{{}^{{}_{t-\Delta t}}}+\varphi_{{}^{{}_{t+\Delta t}}})\\ \underset{M_{f}^{0}}{\underset{︸}{\left(\frac{1}{\Delta t^{2}}M_{f}\right)}}\varphi_{{}^{{}_{t+\Delta t}}}=-\underset{{\hat{F}}_{f}}{\underset{︸}{K_{f}u_{t}}}+\underset{M_{f}^{1}}{\underset{︸}{\left(\frac{2}{\Delta t^{2}}M_{f}\right)}}\varphi_{t}-\underset{M_{f}^{2}}{\underset{︸}{\left(\frac{1}{\Delta t^{2}}M_{f}\right)}}\varphi_{{}^{{}_{t-\Delta t}}}+\underset{C_{f}^{0}}{\underset{︸}{\left(\frac{1}{2\Delta t}C_{f}\right)}}(u_{{}^{{}_{t-\Delta t}}}-u_{{}^{{}_{t+\Delta t}}}). \end{array}$$

The subscripts related to the time *t* and time step Δ*t* define the varying times in the numerical calculation. Two difficulties are implicated in solving the above equations directly: (1) the large dimension of the stiffness matrix and (2) the iteration between two mediums in different time steps.

To simulate wave propagation accurately, the grid should satisfy the rule that at least 10 nodes are required for each wavelength [21,22,23]. This requirement usually involves a large dimensional grid for high-frequency wave motion. On the other hand, a dense grid further compels the use of a tiny Δ*t* (usually less than 1 × 10^−6^ s for ultrasound wave simulation) in the central difference time integration scheme, in order to ensure convergence. In each time step, the matrix calculation and the inverse computation should be iteratively solved. Therefore, Equation (45) is difficult to solve on the level of the global matrix. We chose the pseudo-parallel method (as shown in Table 1) to address this problem.

Secondly, the iteration between fluid and solid is also a key issue for partitioned approaches. Considering the last terms on the right-hand side of Equation (45): (a) in the solid region, in order to estimate the displacement field $u_{{}^{{}_{t+\Delta t}}}$, the distribution of the potential function $\varphi_{{}^{{}_{t+\Delta t}}}$ is required. However, $\varphi_{{}^{{}_{t+\Delta t}}}$ is unknown for the current step; and (b) we can obtain $\varphi_{{}^{{}_{t+\Delta t}}}$ from the fluid region to solve the issue. In the fluid region, the analogous obstacle is that $u^{{}_{t+\Delta t}}$ is unknown. This problem can be addressed by using the partitioned approach based on Jacobi iteration [12], as shown in Table 2.

Based on the definition in Equation (45), the algorithms in Table 1 and Table 2 are proposed. The presented method is a hybrid of the pseudo-parallel method and the partitioned approach, where these two methods are employed to address the memory requirement and iteration problems, respectively. It can be observed that the global stiffness matrix **K**, a semi-full matrix, is not used in the calculation; instead, it is established on an elementary level by the equations ${\hat{f}}_{s}^{e}=K_{s}^{e}u_{t}^{}$ and ${\hat{f}}_{f}^{e,p}=K_{f}^{e}\varphi_{t}^{}$. Thereafter, the matrix assembly is accomplished by ${\hat{f}}_{s}^{}=$$\sum_{e}{\hat{f}}_{s}^{e}$ and ${\hat{f}}_{f}^{}=$$\sum_{e}{\hat{f}}_{f}^{e}$ on a vector level. Based on the above processes, the memory requirement problem is addressed, as the direct multiplication and inversion of global matrices are avoided. Although the mass matrix **M** and interface matrix **C** are assembled in our process, the mass matrix **M** is a diagonal matrix, which can be treated as a vector, and its inverse can be easily calculated (using the reciprocal of each entry). The interface matrix **C** is similar to **M** but sparser. The presented calculations are all accomplished in only one grid system, where the absence of a staggered grid guarantees the simplicity of the structure of the developed algorithm.

## 3. Numerical Examples

Numerical validations were organized in the following manner:

(1) *Cases A–C* were designed for different regions (i.e., from rectangular to solid circle) and different interfaces (i.e., from straight to curved).
(2) Two sub-cases were set in each case (e.g., *Case A*-1 and *Case A*-2 for *Case A*). These two sub-cases were used to simulate wave propagation from solid to fluid and fluid to solid, respectively.
(3) *Cases A*–*C* only present some qualitative comparisons with the theoretical wavefront in snapshots. Quantitative analyses are given at the end of this section, in terms of convergence analysis.
(4) Considering the similarity of *Cases A–C*, convergence analysis was only conducted for *Case C*, the most complex case among them.

### 3.1. Case A: Rectangular with a Straight Interface

**Figure 2:**
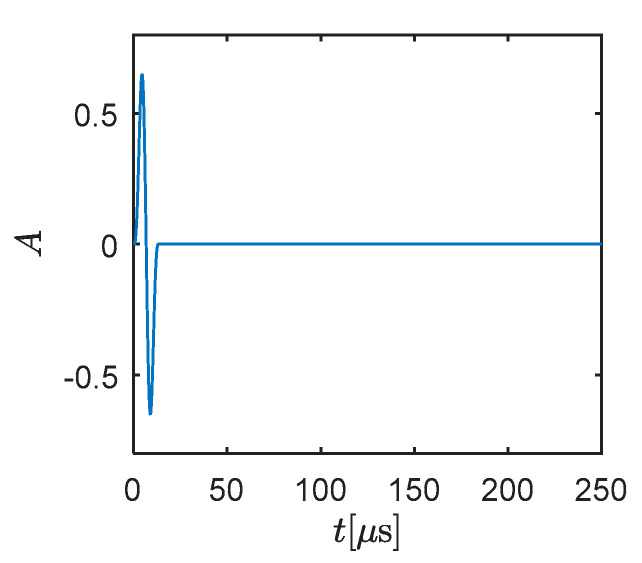
The excitation used in simulations.

In order to demonstrate the performance of the presented method, a simple fluid–solid coupling example is conducted in this section. The inspected regions are treated as the plane strain problem and the material parameters are given in Table 3. In the following analysis, the primary wave (P-wave) and the secondary wave (S-wave) appear. The P-wave is a compressional wave which can move through both solid and fluid. The particles in the medium that the P-wave passes through are pushed and pulled by wave propagation. It travels faster than the S-wave. The S-wave can only move through the solid, not through any liquid medium (as it is a kind of shear wave). Particles are moved back and forth perpendicularly to the direction of wave motion. Due to the assumption of the present fluid medium, only the P-wave exists (1468.63 m/s) in the fluid part. The excitation is shown in Figure 2, which was defined by a modulated wave with the carrier frequency *f*_1_ = 75 kHz and the modulation frequency *f*_2_ = 75 kHz:

(46) $$S=0.5\sin(2\pi f_{1}t)\left[1-\cos(2\pi f_{2}t)\right].$$

The wave propagation is illustrated by contours in the following parts. In the solid region, the contours represent the displacement defined by $\sqrt{u_{x}^{2}+u_{y}^{2}}$, while the contours in the fluid part are drawn based on |φ|. These contours are all normalized, for the sake of expression.

The solid part, with dimensions 0.125 m × 0.25 m, was divided by 10 × 20 BSWI elements—20,301 nodes and 40,602 dofs (degrees of freedom)—and the fluid part, with dimensions 0.125 m × 0.25 m, was also meshed by 10 × 20 BSWI elements—20,301 nodes and 20,301 dofs. It should be noted that the BSWI elements used in this paper had 121 nodes per element, thus having 121 dofs and 242 dofs for fluid and solid, respectively. The number of dofs seemed to be not large enough, compared with some commercial software; however, it becomes further multiplied when time iteration (as shown in Table 1 and Table 2) is considered. For each time step, enough memory was required to compute 60,903 dofs. Usually, more than 5000 steps are used in a typical simulation. The situation can be further compounded when a more complex shape and interface are investigated.

#### 3.1.1. Case A-1: Wave Travels from Solid to Fluid

**Figure 3:**
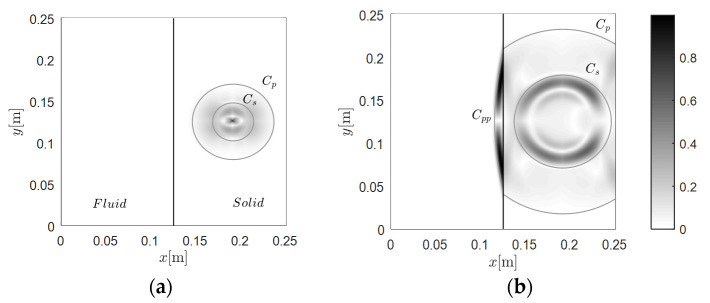

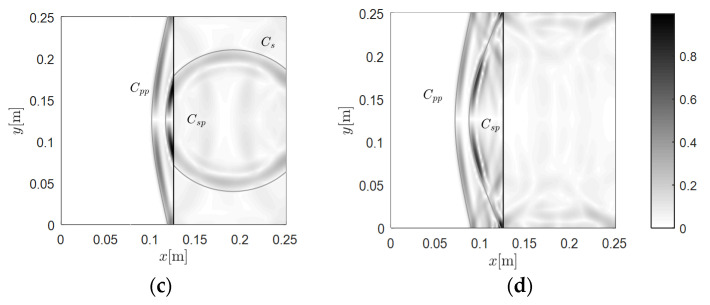
Snapshots of the wavefield induced by the source at 0.191 m and 0.125 m in the solid region: (**a**) 10 μs, (**b**) 20 μs, (**c**) 30 μs, and (**d**) 50 μs.

**Figure 4:**
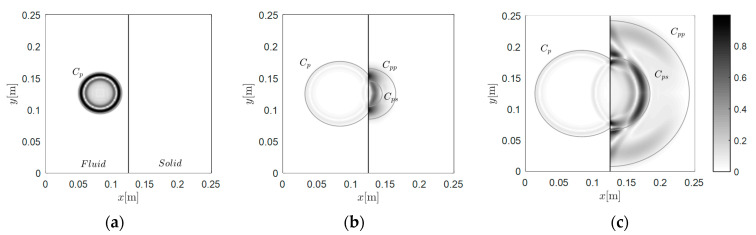
Snapshots of the wavefield induced by the source at 0.083 m and 0.125 m in the fluid region: (**a**) 25 μs, (**b**) 37.5 μs, and (**c**) 50 μs.

Figure 3 presents four snapshots, from 10 to 50 μs, induced by a fixed source located at 0.191 m and 0.125 m in the solid region. The grey auxiliary curves present the theoretical wavefronts of the P-wave *C_p_*, the S-wave *C_s_*, and the secondary wavefronts. The auxiliary solid line located in the middle of the region depicts the interface. The symbol *C_pp_* depicts the P-wave in fluid induced by the P-wave in the solid, and *C_sp_* depicts the P-wave in fluid induced by the S-wave in the solid. In the same manner, we can define the secondary wavefront *C_ps_*, which is presented in Figure 4. One can observe, from Figure 3, that the developed results qualitatively agreed with the wavefronts, either for the main modes *C_p_* and *C_s_*, or the secondary waves *C_pp_* and *C_sp_*. The interface transformed the P- and S-waves in the solid region to the corresponding secondary P-waves in fluid, where the wavefronts are also clear.

#### 3.1.2. Case A-2: Wave Travels from Fluid to Solid

To bilaterally validate the effectiveness of the interface, the source was then moved to the fluid region for verification. The grid was kept the same. In Figure 4, the source was further implemented in the fluid region (at 0.083 m and 0.125 m), and the snapshots were recorded from 25 to 50 μs. Likewise, the numerical P-wave wavefront in fluid qualitatively agreed with the theoretical wavefront. When the wave passed through the interface, two wave modes were generated in the solid region (i.e., *C_pp_* and *C_ps_*). The wavefronts of these two secondary waves also qualitatively agreed with the theoretical wavefronts. The above results validate the effectiveness of the present method in normalized regions.

### 3.2. Case B: Rectangular with a Curved Interface

**Figure 5:**
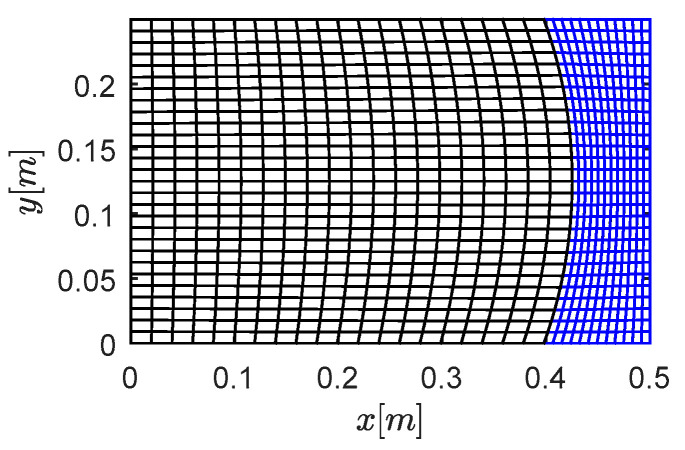
Grids investigated with curved interfaces: rectangular region divided by a curved interface.

**Figure 6:**
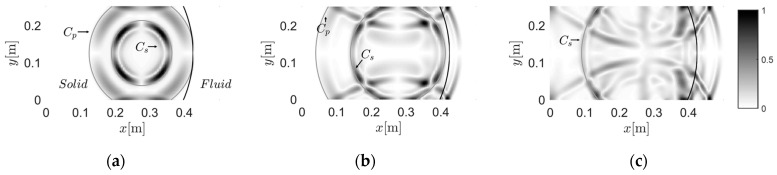
Snapshots of the wavefield induced by the source at 0.275 m and 0.125 m in the solid region: (**a**) 25 μs, (**b**) 37.5 μs, and (**c**) 50 μs.

**Figure 7:**
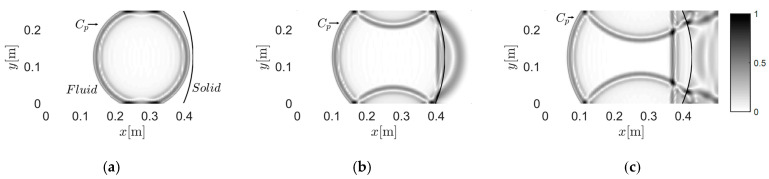

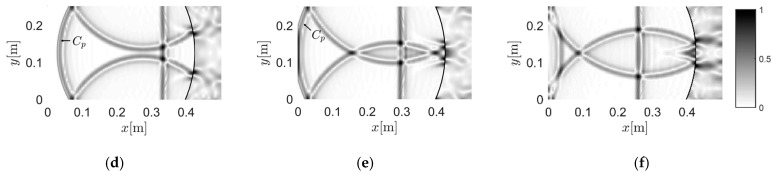
Snapshots of the wavefield induced by the source at 0.275 m and 0.125 m in the fluid region: (**a**) 50 μs, (**b**) 75 μs, (**c**) 100 μs, (**d**) 125 μs, (**e**) 150 μs, (**f**) 175 μs, and (**g**) 200 μs.

Curved interfaces and boundaries are further investigated in this section. The material is presented in Table 4. The grid of *Case B*, a rectangular region divided by a curve, is shown in Figure 5. The left part was meshed by 560 wavelet elements (56,481 nodes) and the right part was meshed by 336 wavelet elements (34,001 nodes). Compared with the uniform grid for solid and fluid used in Figure 3 and Figure 4, the mesh in Figure 5 was intentionally designed with different dimensions. As mentioned, the verification was hoped to be bidirectional. Thus, the left grid was used to model the solid region in *Case B-*1 (as shown in Figure 6), and then to model the fluid region in *Case B*-2 (as shown in Figure 7).

#### 3.2.1. Case B-1: Wave Travels from Solid to Fluid

Figure 6 presents the wave propagation from solid to fluid. The left part was treated as solid and, thus, had 112,962 dofs; while the grid on the right of the interface was used to simulate the fluid, with 34,001 dofs. The source was fixed at 0.275 m and 0.125 m in the solid region. Due to the complexity of the theoretical secondary wavefronts, we only present the main modes in the following simulations. Figure 6a shows the overall view of the P-wave and S-wave, whose wavefronts qualitatively agreed with the theoretical ones. In Figure 6b, the P-wave passes through the interface and a clear secondary P-wave in the fluid region is generated after 37.5 μs. Figure 6c presents the transition from S-wave to the corresponding secondary P-wave in the fluid part after 50 μs. It can be seen that the snapshot is complex and blurred, due to reflections.

#### 3.2.2. Case B-2: Wave Travels from Fluid to Solid

In the following simulation, shown in Figure 7, we exchange the solid and fluid grids, such that the left part was defined as fluid, with 56,481 dofs; while the right part was defined as solid, with 68,002 dofs. The source was still fixed at 0.275 m and 0.125 m, but was in the fluid region. Figure 7a illustrates the original P-wave. Figure 7b,c show the generation of the secondary waves when the original P-wave passes through the interface. As the source is near the focus of the interface, the reflection from the interface is nearly straight. The plane wave travels to the left and its shape remains as a line before 200 μs, as shown in Figure 7d–f.

### 3.3. Case C: Solid Circle with a Curved Interface

**Figure 8:**
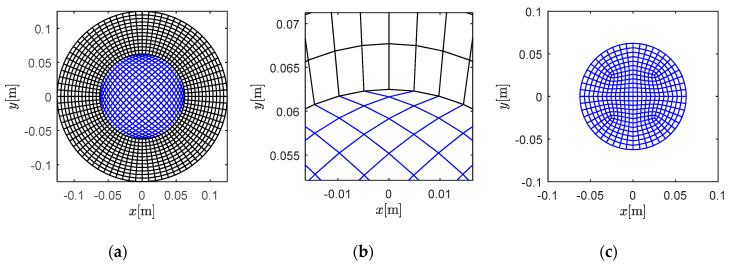
Grids investigated with curved interfaces: (**a**) the solid circular region divided by a curved interface, (**b**) detailed view of non-optimal mesh area, and (**c**) an alternative grid mode for the inner region.

In *Case C* (Figure 8), a circular region with a curved interface was considered. The annulus was defined as the fluid region (960 elements, 160,800 dofs), while the inner circle was defined as the solid region (400 elements, 80,802 dofs). The grid in Figure 8a is not optimized, in view of the finite element method, as some elements nearly degenerate to triangles, as shown in Figure 8b. This means that the Jacobi matrix is nearly singular here. Furthermore, the dimension of the elements in the inner part of Figure 8a are not uniform, which involves the potential instability in temporal discretization and solution. Figure 8c presents an alternative grid mode for the inner region, with more uniform dimension and shape. However, in this section, we chose the worse grid for validation, based on the idea that, if the method performs well on a bad grid, it could perform well on better meshes.

#### 3.3.1. Case C-1: Wave Travels from Solid to Fluid

**Figure 9:**
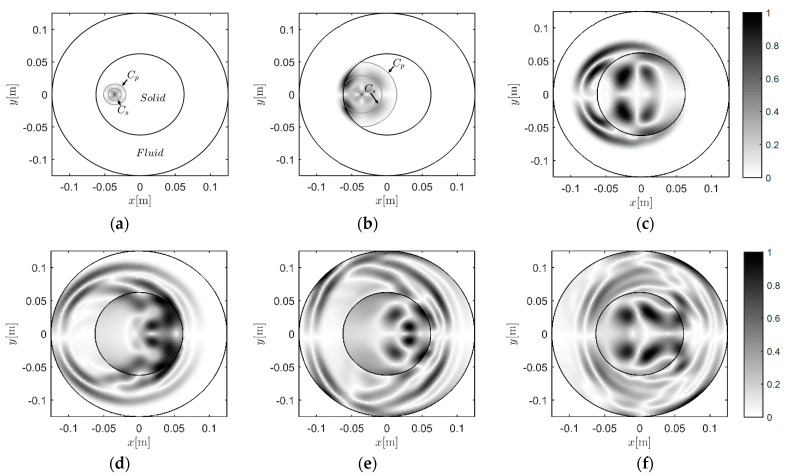
Snapshots of the wavefield induced by the source at −0.0360 m, and 0 m: (**a**) 20 μs, (**b**) 25 μs, (**c**) 35 μs, (**d**) 45 μs, (**e**) 55 μs, and (**f**) 65 μs.

Figure 9 shows the wave propagating from solid to fluid in the inspected region. Figure 9a,b present the early development of the wave. The agreement between the theoretical wavefront and the numerical wavefront validated the effectiveness of the method in the solid region. In the snapshot at 25 μs (Figure 9b), the P-waves in fluid induced by the P- and S-waves in solid are concentrated near the interface. In Figure 9c–e, the secondary P-waves in fluid induced by the P- and S-waves in solid can be observed and distinguished. In addition, the presented result maintained the symmetry of the wavefield, as can be seen in Figure 9f.

#### 3.3.2. Case C-2: Wave Travels from Fluid to Solid

**Figure 10:**
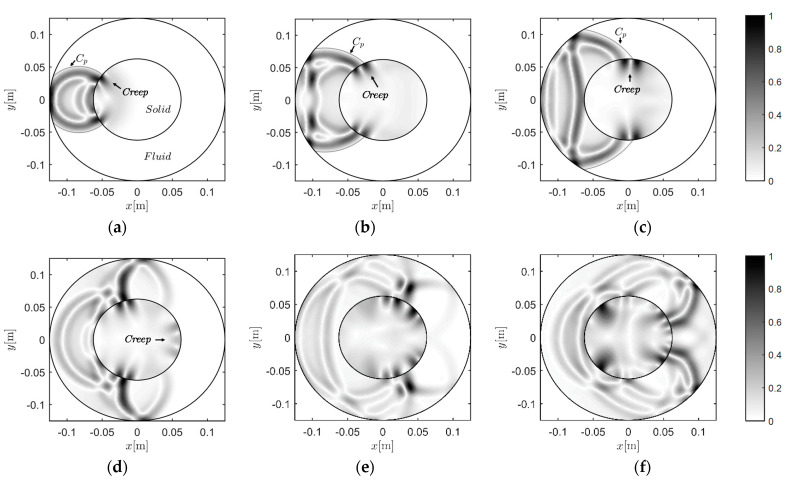
Snapshots of the wavefield induced by the source at −0.0833 m and 0 m: (**a**) 35 μs, (**b**) 45 μs, (**c**) 55 μs, (**d**) 75 μs, (**e**) 95 μs, and (**f**) 115 μs.

Then, the source is further replaced at −0.0833 m and 0 m in the fluid region. The corresponding snapshots are given in Figure 10. We can see that creep waves were generated, due to the shape of the interface. The creep wave travelled along the interface, following it in a curved manner (as shown in Figure 10d–f). Induced by the creep wave, wave motions occurred in the area shadowed by the solid region.

### 3.4. Convergence Analysis in Space

Convergence analyses were conducted on *Case C* for three types of grid:

(1) Grid-1, the densest mesh composed of 1920 elements (321,600 dofs) for the fluid part and 800 solid elements (161,604 dofs) for the solid part.
(2) Grid-2, the grid used in Section 3.3, which was composed of 960 elements (160,800 dofs) for the fluid part and 400 solid elements (80,802 dofs) for the solid part.
(3) Grid-3, a denser mesh composed of 648 elements (66,744 dofs) for the fluid part and 324 solid elements (66,746 dofs) for the solid part.
(4) Grid-4, a sparse mesh composed of 392 elements (40,448 dofs) for the fluid part and 196 solid elements (40,490 dofs) for the solid part.

It should be mentioned that the grids were similar in structure, but had different densities.

**Figure 11:**
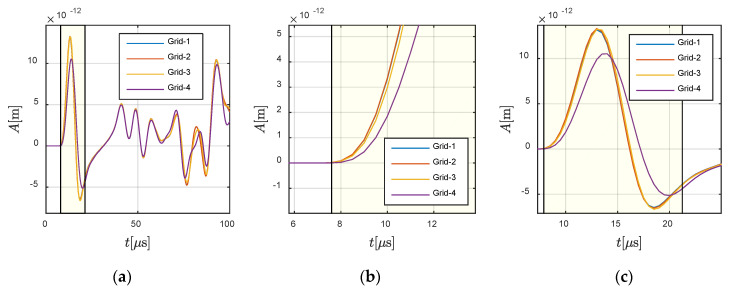
Responses in X-direction at −0.0625 m and 0 m in the solid region: (**a**) global view and (**b**,**c**) local views. The highlighted part defines the theoretical wavefront and duration of the P-wave.

**Figure 12:**
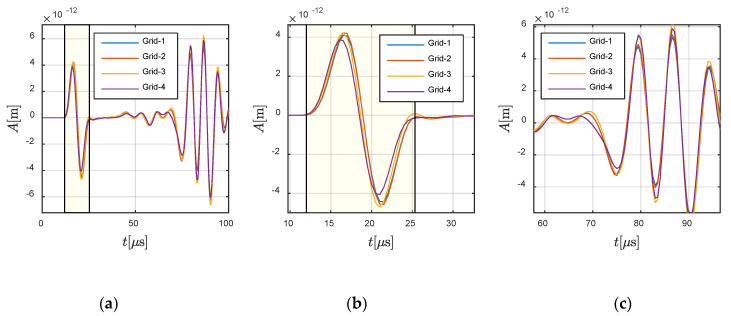
Responses in Y-direction at −0.0625 m and 0 m in the solid region: (**a**) global view and (**b**,**c**) local views. The highlighted part defines the theoretical wavefront and duration of S-wave.

First, convergence analyses were conducted on *Case C*-1, in which the wave travelled from the solid part to the fluid part. The wave source was located at −0.0360 m and 0 m, and assigned vibrations along X-direction. The receiver was located at −0.0625 m and 0 m in the solid region. The fluctuation of the wave source determined that the X-direction responses at the receiver were dominated by the P-wave (as shown in Figure 11), and that the Y-direction here was dominated by the S-wave (as shown in Figure 12).

**Figure 13:**
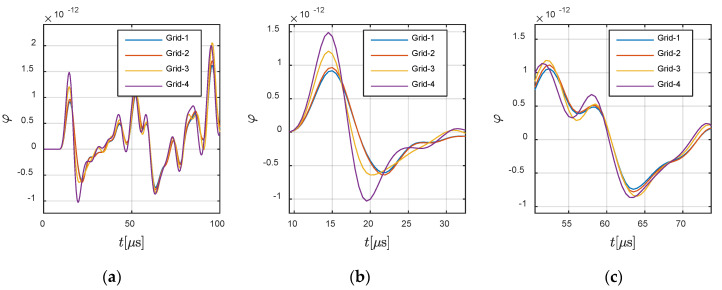
Responses at −0.0700 m and 0 m in the fluid region: (**a**) global view and (**b**,**c**) local views.

In order to illustrate the P- and S-waves clearly, the X- and Y-directional responses are shown in Figure 11 and Figure 12, respectively. The highlighted part in Figure 11 defines the theoretical wavefront and duration of P-wave. It can be observed that the presented results, for all grids, agreed with the theoretical wavefront. A similar conclusion can be obtained from Figure 12 for the S-wave, in which the highlighted part defines the corresponding parameters for S-wave. Meanwhile, the fluctuation in the fluid at (−0.0700 m, 0 m) is presented in Figure 13, in order to demonstrate the convergence of the method. It can be seen that the different grids achieved convergence in the fluid part.

**Figure 14:**
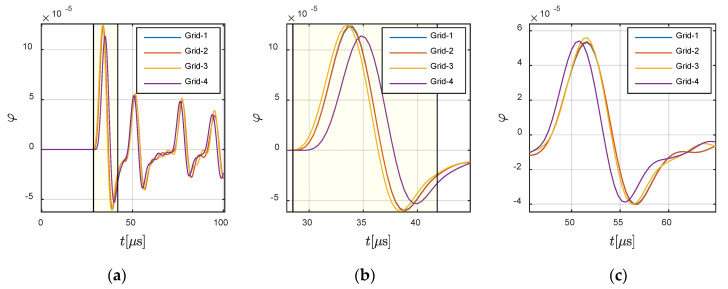
Responses at −0.125 m and 0 m in the fluid region: (**a**) global view and (**b**,**c**) local views. The highlighted part defines the theoretical wavefront and duration of the P-wave.

**Figure 15:**
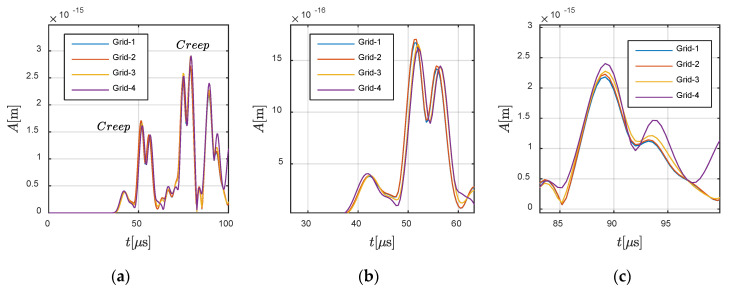
Responses at 0 m and 0.0625 m in the solid region: (**a**) global view and (**b**,**c**) local views.

Further analysis was conducted on *Case C-2*, in which the wave travelled from fluid to solid. The source was located at −0.0833 m and 0 m, and the receiver was assigned at −0.125 m and 0 m in the fluid and at 0 m and 0.0625 m in the solid. Figure 14 and Figure 15 show the convergence in the fluid part and solid part, respectively. For a better illustration of the creep wave, the response in the solid part is presented in the form of $\sqrt{u_{x}^{2}+u_{y}^{2}}$, keeping consistency with the snapshots shown in Figure 10. It can be observed that the present method also converged for *Case C-2*.

### 3.5. Convergence Analysis in Time Domain

**Figure 16:**
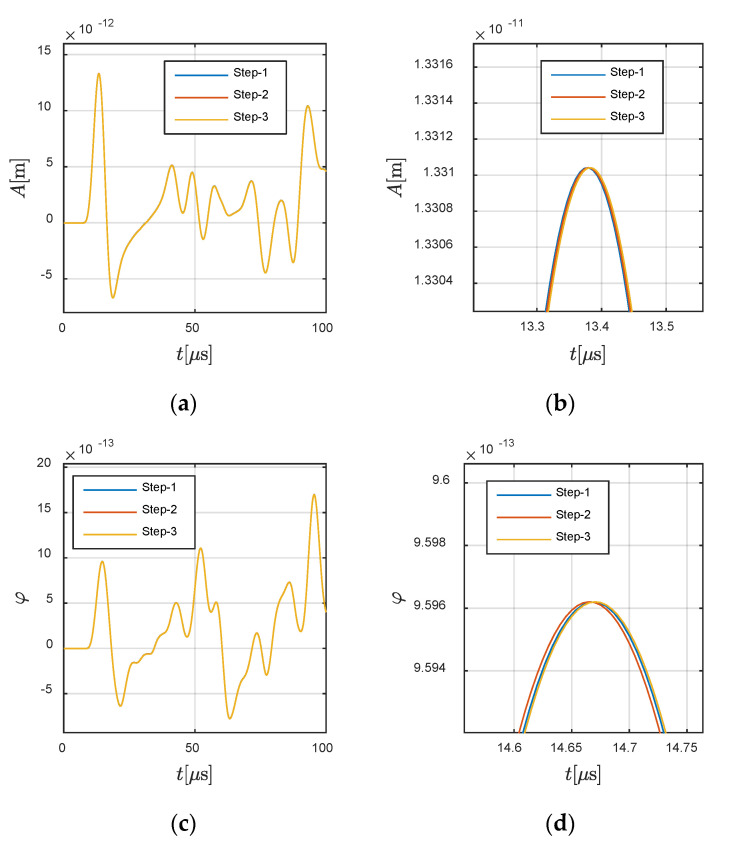
Convergence analysis in time domain: (**a**,**b**) in solid region, (**c**,**d**) in fluid region. (**b**,**d**) are the local views of (**a**,**b**).

Besides the convergence analysis on space, the convergence analysis in time domain is also conducted in this section based on Gird-2. In the premise of avoiding the numerical diffusion in central difference time integration scheme, we vary the time step for presentation. Time steps are selected as 0.01 μs, 0.005 μs, and 0.0025 μs, denoted by “Step-1” to “Step-3”, respectively. Figure 16 presents the results in solid and fluid. Compared with the convergence in space, the influence induced by the change of time step is tiny if it satisfies the convergency requirement of central difference convergence.

## 4. Discussions about Relaxation

As presented in Table 1, the relaxation method was not used; however, this does not mean that relaxation is not required in the present method. Thus, a discussion of the convergence of the partitioned approach is given in this section. The general formulation can be described by:

(47) $$\omega^{k}\varphi^{k+1}+(1-\omega^{k})\varphi^{k}\to \varphi^{k+1}.$$

The weighting constant *ω* is also called the relaxation parameter. The simplest method is to choose a fixed parameter ω for all time steps, in which the relaxation parameter has to be small enough to keep the iteration from diverging, but as large as possible to avoid unnecessary iterations. In addition, non-relaxation results can be obtained by setting *ω* = 1. Besides the fixed method, some self-adaptive relaxation methods are also available, such as Aitken relaxation [24]. The relaxation parameter is calculated by Equations (48) and (49) in the Aitken relaxation method:

(48) $$r^{k+1}=\varphi^{k+1}-\varphi^{k},$$

(49) $$\omega^{k}=-\omega^{k}\frac{{\left(r^{k}\right)}^{T}(r^{k+1}-r^{k})}{{\left(r^{k+1}-r^{k}\right)}^{T}(r^{k+1}-r^{k})}.$$

These relaxation methods can be integrated into the present method (see Table 2). To demonstrate the performance of these methods, the 1st–250th time steps of *Case B*-1 were selected. The convergence criteria *e_s_* and *e_f_* (see Table 1) were used as inputs and the average number of iterations (denoted by “*i*” in Table 5) was defined as the output.

**Figure 17:**
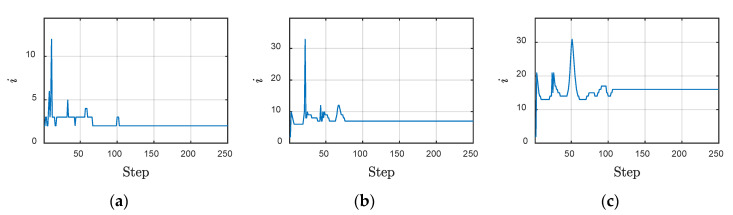
The convergence behavior of Aitken relaxation: (**a**) *e_s_* = 10^−13^, *e_f_* = 10^−13^; (**b**) *e_s_* = 10^−14^, *e_f_* = 10^−14^; and (**c**) *e_s_* = 10^−15^, *e_f_* = 10^−15^.

It is clear that Aitken relaxation performed best among the inspected methods. From the convergence behavior of Aitken relaxation shown in Figure 17, one can observe that the iteration steps became stable swiftly after 100 steps, which is nearly the end instant of the wave source. These results demonstrate the adaptability of the Aitken method. By contrast, the average number of iterations for fixed relaxation methods were all linear with the value shown in Table 4 and, hence, are not presented here. For a simple problem such as that considered in this paper, even the non-relaxation method (*ω* = 1) can achieve a satisfying iteration speed, while under-relaxations converge slowly.

## 5. Conclusions

In this work, the inviscid fluid–solid coupling problem was solved by an iterative model using the wavelet element method and a partitioned approach. Based on the use of the Jacobian matrix in the wavelet element method, problems relating to curved boundaries can be solved. The interface was integrated into the model as a pseudo-damping layer, which can conduct transformations between force vectors in the solid region and pressure in the inviscid fluid region. As validated by our results, the presented method can serve as an alternative choice for the issue under consideration, both for normal regions and the curved region.

This work focused on inviscid fluid, but the problems relating to the influence of viscosity on the interface and numerical format (e.g., the implementation of boundary conditions and the iteration method used) can be further considered and investigated. To achieve efficient computation, fixed grid technology was used for the fluid region, by use of a Cartesian grid. As a result, the flow solver may be simple and fast; however, it sacrifices accuracy near the interface, due to the interpolations used. The use of a finite element method in both solid and fluid regions makes the formulation simple; however, the accuracy depends on the ratio between the fluid and solid grid size. Due to the effects of temporal iteration and numerical oscillation on convergence behavior, the sizes of elements are determined by the highest frequency domain considered, which may necessitate a memory- and time-related computational burden.

## Figures and Tables

**Table 1:** Main Algorithm.

Main Algorithm
**#1 Loop over elements *e*** Calculate the elemental matrices **K*_s_^e^***, **M*_s_^e^***, **C*_s_^e^*** and **K*_f_^e^***, **M*_f_^e^***, **C*_f_^e^*** Assemble mass matrices **M*_s_***, **M*_f_***, interface matrices **C*_s_***, **C*_f_***, and vector ***f_t_*** Store all elemental stiffness matrices **K*_s_^e^*** and **K*_f_^e^*** **#1 End of the loop over element *e*** Calculate the auxiliary vectors $M_{s}^{0}$, $M_{s}^{1}$, $M_{s}^{2}$, $C_{s}^{0}$ and $M_{f}^{0}$, $M_{f}^{1}$, $M_{f}^{2}$, $C_{f}^{0}$ Apply the initial condition Initialization, let $u_{t+\Delta t}^{u}=u_{{}^{{}_{t-\Delta t}}}$, $\varphi_{{}^{{}_{t+\Delta t}}}^{u}=\varphi_{{}^{{}_{t-\Delta t}}}^{}$ **#2 Loop over *t*** While ${\left\|u_{{}^{{}_{t+\Delta t}}}^{u}-u_{{}^{{}_{t+\Delta t}}}^{p}\right\|}_{2}\geq e_{s}$ or ${\left\|\varphi_{{}^{{}_{t+\Delta t}}}^{u}-\varphi_{{}^{{}_{t+\Delta t}}}^{p}\right\|}_{2}\geq e_{f}$ Invoke “**The partitioned approach based on Jacobi iteration**” End **#2 End of the loop over instant *t*** Output $u=u_{{}^{{}_{}}}^{u}$, $\varphi =\varphi_{{}^{{}_{}}}^{u}$

The superscript “*u*” represents the “updating step”. The superscript “*p*” represents the “predicting step”.

**Table 2:** The partitioned approach based on Jacobi iteration.

The Partitioned Approach Based on Jacobi Iteration
Calculate ${\hat{f}}_{s}^{e}=K_{s}^{e}u_{t}^{}$ and ${\hat{f}}_{f}^{e,p}=K_{f}^{e}\varphi_{t}^{}$ Assemble vectors ${\hat{f}}_{s}^{}=$$\sum_{e}{\hat{f}}_{s}^{e}$ and ${\hat{f}}_{f}^{}=$$\sum_{e}{\hat{f}}_{f}^{e}$ Calculate force vectors $\begin{array}{c} {\tilde{R}}_{s}^{p}=f_{t}-{\hat{f}}_{s}^{}+M_{s}^{1}u_{t}-M_{s}^{2}u_{t-\Delta t}+C_{s}^{0}(\varphi_{{}^{{}_{t-\Delta t}}}+\varphi_{{}^{{}_{t+\Delta t}}}^{u}) \end{array}$ and $\begin{array}{c} {\overset{-}{R}}_{f}^{p}=-{\hat{f}}_{f}^{}+M_{f}^{1}\varphi_{t}-M_{f}^{2}\varphi_{t-\Delta t}+C_{f}^{0}(u_{{}^{{}_{t-\Delta t}}}-u_{t+\Delta t}^{u}) \end{array}$ Predict the vectors $u_{{}^{{}_{t+\Delta t}}}^{p}={\left(M_{s}^{0}\right)}^{-1}{\overset{-}{R}}_{s}^{p}$ and $\varphi_{{}^{{}_{t+\Delta t}}}^{p}={\left(M_{f}^{0}\right)}^{-1}{\overset{-}{R}}_{f}^{p}$ Calculate force vectors ${\tilde{R}}_{s}^{u}=f_{t}-{\hat{f}}_{s}^{}+M_{s}^{1}u_{t}-M_{s}^{2}u_{t-\Delta t}+C_{s}^{0}(\varphi_{{}^{{}_{t-\Delta t}}}+\varphi_{{}^{{}_{t+\Delta t}}}^{p})$ and ${\overset{-}{R}}_{f}^{u}=-{\hat{f}}_{f}^{}+M_{f}^{1}\varphi_{t}-M_{f}^{2}\varphi_{t-\Delta t}+C_{f}^{0}(u_{{}^{{}_{t-\Delta t}}}-u_{t+\Delta t}^{p})$ Update the vectors $u_{{}^{{}_{t+\Delta t}}}^{u}={\left(M_{s}^{0}\right)}^{-1}{\overset{-}{R}}_{s}^{p}$ and $\varphi_{{}^{{}_{t+\Delta t}}}^{u}={\left(M_{f}^{0}\right)}^{-1}{\overset{-}{R}}_{f}^{p}$ Relaxation (alternative): $u_{{}^{{}_{t+\Delta t}}}^{u}=\omega u_{{}^{{}_{t+\Delta t}}}^{u}+(1-\omega )u_{{}^{{}_{t+\Delta t}}}^{p}$ $\varphi_{{}^{{}_{t+\Delta t}}}^{u}=\omega \varphi_{{}^{{}_{t+\Delta t}}}^{u}+(1-\omega )\varphi_{{}^{{}_{t+\Delta t}}}^{p}$

The superscript “*u*” represents the “updating step”. The superscript “*p*” represents the “predicting step”. *ω* denotes the relaxation parameter, see Section 4.

**Table 3:** Parameters of the regular regions.

Medium	Modulus/GPa	Density/kg·m^−3^	Poisson’s Ratio	*c_p_*/m·s^−1^	*c_s_*/m·s^−1^
Solid	70.0	2700	0.33	6197.82	3121.75
Fluid	-	1020	-	1468.63	-

**Table 4:** Parameters of the curved regions.

Medium	Modulus/GPa	Density/kg·m^−3^	Poisson’s Ratio	*c_p_*/m·s^−1^	*c_s_*/m·s^−1^
Solid	25.6	2500	0.21	3395.10	2057.00
Fluid	-	1020	-	1468.63	-

**Table 5:** Convergence comparisons of relaxation.

Convergence Criterion		Average Number of Iterations				
*e_s_*	*e_f_*	*ω* = 1	*ω* = 0.98	*ω* = 0.90	*ω* = 0.60	Aitken
1 × 10^−10^	1 × 10^−10^	1	1	1	1	1
1 × 10^−13^	1 × 10^−13^	4	12	19	44	2.328
1 × 10^−14^	1 × 10^−14^	9	15	20	47	7.360
1 × 10^−15^	1 × 10^−15^	16	18	21	49	16.892
